# Tick-borne encephalitis virus transmitted singly and in duo with *Borrelia burgdorferi *sensu lato and *Anaplasma phagocytophilum* bacteria by ticks as pathogens modifying lipid metabolism in human blood

**DOI:** 10.1186/s12929-024-01016-7

**Published:** 2024-03-04

**Authors:** Marta Dobrzyńska, Anna Moniuszko-Malinowska, Piotr Radziwon, Sławomir Pancewicz, Agnieszka Gęgotek, Elżbieta Skrzydlewska

**Affiliations:** 1 Department of Analytical Chemistry, Medical University of Bialystok, Mickiewicza 2D, 15-222 Bialystok, Poland; 2 Department of Infectious Diseases and Neuroinfections, Medical University of Bialystok, Zurawia 14, 15-540 Bialystok, Poland; 3 Regional Centre for Transfusion Medicine, M. Sklodowskiej-Curie 23, 15-950 Bialystok, Poland

**Keywords:** TBE, *Borrelia burgdorferi *sensu lato, *Anaplasma phagocytophilum*, Lipid metabolism, Lipidomics

## Abstract

**Background:**

Ticks are vectors of various pathogens, including tick-borne encephalitis virus causing TBE and bacteria such as *Borrelia burgdorferi *sensu lato and *Anaplasma phagocytophilum* causing e.g. viral-bacterial co-infections (TBE + LB/HGA), which pose diagnostic and therapeutic problems. Since these infections are usually accompanied by inflammation and oxidative stress causing metabolic modifications, including phospholipids, the aim of the study was to assess the level of polyunsaturated fatty acids and their metabolism (ROS- and enzyme-dependent) products in the blood plasma of patients with TBE and TBE + LB/HGA before and after pharmacotherapy.

**Methods:**

The total antioxidant status was determined using 2,20-azino-bis-3-ethylbenzothiazolin-6-sulfonic acid. The phospholipid and free fatty acids were analysed by gas chromatography. Lipid peroxidation was estimated by measuring small molecular weight reactive aldehyde, malondialdehyde and neuroprostanes. The reactive aldehyde was determined using gas chromatography coupled with mass spectrometry. The activity of enzymes was examined spectrophotometrically. An analysis of endocannabinoids and eicosanoids was performed using a Shimadzu UPLC system coupled with an electrospray ionization source to a Shimadzu 8060 Triple Quadrupole system. Receptor expression was measured using an enzyme-linked immunosorbent assay (ELISA).

**Results:**

The reduced antioxidant status as a result of infection was accompanied by a decrease in the level of phospholipid arachidonic acid (AA) and docosahexaenoic acid (DHA) in TBE, an increase in DHA in co-infection and in free DHA in TBE with an increase in the level of lipid peroxidation products. The enhanced activity of enzymes metabolizing phospholipids and free PUFAs increased the level of endocannabinoids and eicosanoids, while decreased 15-PGJ2 and PGE2 was accompanied by activation of granulocyte receptors before pharmacotherapy and only tending to normalize after treatment.

**Conclusion:**

Since classical pharmacotherapy does not prevent disorders of phospholipid metabolism, the need to support treatment with antioxidants may be suggested.

## Introduction

According to the World Health Organization, infectious diseases pose a huge threat to human health, accounting for around 17% of all deaths worldwide in 2020. Ticks are vectors of a wide spectrum of pathogens that carry viruses including e.g. tick-borne encephalitis virus (TBEV) belonging to the Flaviviridae family as well as bacteria such as *Borrelia burgdorferi *sensu lato and *Anaplasma phagocytophilum* [1], which cause diseases that pose epidemiological and clinical problems. It is also known that the *Ixodes ricinus* tick can transmit several pathogens at the same time, of which, apart from TBEV, the most prevalent are *B. burgdorferi *s.l. and *A. phagocytophilum* [2]. This may lead to co-infections, which are even more difficult to diagnose and treat due to non-specific symptoms.

Tick-borne diseases are a serious medical problem for diagnostic reasons because they can have forms with unspecific manifestations like fever, chills, headache, weakness, nausea and vomiting [3]. However, regardless of the clinical manifestation, these diseases are characterized by metabolic disorders associated with inflammation and disturbed redox balance [4], which is disrupted, among others, by as a result of overexpression of the NS1 protein in response to the presence of TBEV, which disrupts the Nrf2/ARE pathway responsible for the biosynthesis of cytoprotective proteins, including antioxidants [5]. The oxidative stress observed in TBE is responsible for the oxidative modifications of cellular components and body fluids, including proteins, lipids and nucleic acids [4, 6], which also favors the modification of the environment of the NFκB transcription factor leading to complex metabolic disorders resulting in pathophysiological changes, including inflammation and exacerbation of the disease process [7]. Both oxidative stress and inflammation are the main elements of the host's immune response to viral infection, which, interacting metabolically with each other, can play both a protective as well as pathogenic role [1].

The structural components of biological membranes are lipids, which are also important bioactive molecules mediating in signal cascades and regulatory events in cells and biological fluids, including response to infections, especially viral ones, because the ability to biosynthesis lipids predisposes the human body to ingest parasites [8]. As obligate parasites, viruses rely solely on host resources to meet their lipid needs In the case of most viruses replicating in the cytoplasm of infected cells, lipids are necessary for the replication of their genomes [8]. In addition, host membrane structures are modified to evade antiviral defense mechanisms, but also to provide a site for viral replication [9, 10].

Recently, it has been shown that tick-borne infections, by promoting increased ROS generation in the plasma and reducing antioxidant defense, especially in relation to the glutathione and thioredoxin systems, what promotes oxidative modifications of phospholipid PUFAs with increased lipid peroxidation (estimated as 8-isoPGF2α, 4-HNE) [4]. This is accompanied by protein modifications that favor the structural and functional modification of granulocytes Nrf2 and NFκB transcription factors responsible for the biosynthesis of antioxidants and pro-inflammatory factors, respectively [4]. This suggests that the consequences of the disease may lead to the development of neurodegenerative disorders, supported by the fact that the neurodegenerative process persists even after the clinical recovery of these patients [11, 12]. In addition, oxidative stress accompanying tick-borne diseases is conducive to increasing the activity of lipolytic enzymes, including PLAs, COXs, LOXs, which metabolize PUFAs to lipid mediators such as endocannabinoids and eicosanoids that are also involved in modeling the redox balance and inflammation in the patient's organism [8]. It has been shown that the development of TBE is accompanied by an increased concentration of sphingosine-1-phosphate (S1P) in the cerebrospinal fluid, which is a product of sphingomyelin metabolism, which is a signaling molecule involved in inflammatory processes and immune reactions and plays a key role in the differentiation, survival and excitability of neurons. It also participates in the repair of neurons by reducing oxidative stress in astrocytes [13]. However, overexpression of triacylglycerols was also observed in the febrile phase of TBE [14].

However, it is also known that the *Ixodes ricinus* tick can transmit several pathogens at the same time, of which, apart from TBEV, the most famous are *B. burgdorferi *s.l. and *A. phagocytophilum* [2]. This may lead to co-infections, which are even more difficult to diagnose and treat due to non-specific symptoms.

Therefore, the relationship between the type of pathogen/pathogens and metabolic changes, including in relation to phospholipids, and the patient's disease state is constantly being sought, hoping to improve the effectiveness of therapeutic solutions related to the possibility of affecting lipid mediators. Therefore, the aim of this study was to compare changes at the level of plasma lipid mediators and activation of granulocyte membrane receptors involved in the regulation of both redox balance and inflammation. The studies were conducted using the blood of patients infected with TBEV alone and patients with bacterial co-infections (*B.* *burgdorferi *s.l. and *A. phagocytophilum*). The studies were conducted using blood collected from patients in the early phase and after treatment.

## Material and methods

### Samples collection

Blood samples were collected from 40 patients with tick-borne encephalitis (TBE), including 14 women and 26 men, with an average age of 40 years (from 23 to 58 years) that were admitted to the Department of Infectious Diseases and Neuroinfections of the Medical University of Bialystok. In addition, blood was collected from 6 patients (including 4 females and 2 males) who had co-infections with other tick-borne pathogens such as *Borrelia burgdorferi *sensu lato (Bb) causing *Lyme borreliosis* (LB) and *Anaplasma phagocytophilum* (Ap) causing human granulocytic anaplasmosis (HGA). According to the European Academy of Neurology (EAN) guidelines [15], TBE was diagnosed based on clinical symptoms, positive serology and lymphocytic pleocytosis in the cerebrospinal fluid (CSF). LB was determined by the presence of erythema migrans (1 patient) or possible neuroborreliosis (1 patient). In addition, in two patients anti-*Borrelia burgdorferi *sensu lato IgM and IgG antibodies were detected and, consequently, the patients were treated according to the standard. However, HGA was diagnosed based on CDC criteria when all 3 criteria were satisfied (https://wwwn.cdc.gov/nndss/conditions/ehrlichiosis-and-anaplasmosis/case-definition). Human granulocytic anaplasmosis was diagnosed in four cases on the basis of clinical picture, laboratory tests and PCR, of which Lyme disease was additionally diagnosed among two people. In summary, triple co-infection (TBE + borreliosis + anaplasmosis) occurred in two patients, double co-infection: TBE + borreliosis was diagnosed among two patients and TBE + anaplasmosis was detected in two patients. Patients with TBE were treated symptomatically (antioedematous drugs, anti-inflammatory drugs, analgesics and glucocorticosteroids), patients with anaplasmosis received doxycycline, while those with Lyme disease received doxycycline or ceftriaxone. Before compiling the results of the manuscript, we analyzed the parameters of patients who were given glucocorticosteroids during treatment and patients who did not receive glucocorticosteroids during treatment. These parameters did not differ significantly between the two groups.

The study was conducted in accordance with the Declaration of Helsinki, and the protocol for the collection of all blood samples was approved by the Local Bioethics Committee in Medical University of Bialystok (Poland), No. R-I-002/169/2018. Written informed consent was obtained from all participants.

The control group consisted of 20 healthy people (7 women and 13 men) with a mean age of 41 (28–55).

Blood from patients was collected twice, once on admission to the hospital and again after treatment. Blood from control group was obtained once. The mean time between these two examinations in patients with TBE was 29.4 ± 10.99 days, while in patients with co-infection it was 32.8 ± 4.21 days. Blood was collected into EDTA tubes and then centrifuged at 3000×*g* (4 °C) to separate the plasma as well as buffy coat, and erythrocytes. To obtain clear fraction of granulocytes, obtained buffy coat was layered on Gradisol G (Aqua-Med ZPAM–KOLASA, Łódź, Poland) and subjected to 25 min centrifugation at 300×*g* at room temperature. The purity of the obtained cell fraction was examined microscopically (Nikon Eclipse Ti, Nikon Instruments Inc., New York, NY, USA). Obtained plasma and granulocytes were stored at − 80 °C and used for analysis.

Tables 1 and 2 contain demographic and clinical data of patients and controls, as well as their laboratory data.

**Table 1:** Demographic and clinical data of patients with TBEV infection (TBE) and patients with TBEV + Bb/Ap co-infection (co-infection) compared to healthy subjects (control)

	Control	TBE	Co-infection
Age (years)	41 (28–55)	40 (23–58)	42 (22–63)
Sex	5/20 female (25%) 15/20 male (75%)	14/40 female (35%) 26/40 male (65%)	4/6 female (67%) 2/6 male (33%)
Noticeable tick bite	0/20 (0%)	21/40 (52%)	5/6 (83%)
Time since tick bite (days)	–	26.8 ± 16.6	17.58 ± 4.95
Duration of hospitalization (days)	–	12.85 ± 2.6	12.34 ± 1.97
Duration of symptoms (days)	–	7.5 ± 6.9	4.26 ± 2.63
Clinical form			
Meningitis	0/20(0%)	25/40 (62.5%)	5/6 (83%)
Meningoencephalitis	0/20 (0%)	15/40 (37.5%)	1/6 (17%)
Meningoencephalomyelitis	0/20 (0%)	0/40 (0%)	0/6 (0%)

**Table 2:** Laboratory data of patients with TBEV infection (TBE) and patients with TBEV + Bb/Ap co-infection (co-infection) compared to healthy subjects (control)

	Normal range	TBE		Co-infection	
		Before treatment [at admission] [TBE1]	After treatment [after recovery] [TBE2]	Before treatment [at admission] [co-infection1]	After treatment [after recovery] [co-infection2]
Complete blood count					
WBC [10^3^/μL]	4.00–10.00	10.16 ± 2.37	6.11 ± 1.48	7.27 ± 1.36	5.37 ± 1.46
Neutrophils [%]	40.0–72.0	73.77 ± 9.52	51.18 ± 7.93	60.33 ± 7.07	42.58 ± 11.58
Lymphocytes [%]	18.00–48.00	16.2 ± 7.76	34.83 ± 6.35	27.37 ± 6.11	42.72 ± 11.24
Monocytes [%]	2.50–10.00	8.99 ± 2.53	9.79 ± 2.15	10.15 ± 2.61	9.56 ± 3.18
RBC [10^6^/μL]	4.00–5.50	4.40 ± 0.43	4.50 ± 0.32	4.28 ± 0.56	4.14 ± 0.62
HGB [g/dL]	12.00–16.00	13.32 ± 1.28	13.68 ± 1.09	12.67 ± 1.26	12.5 ± 1.39
PLT [10^3^/μL]	130–350	251.44 ± 87.43	270.59 ± 134.4	262.67 ± 66.98	218.4 ± 19.42
CRP [mg/L]	0.00–5.00	11.52 ± 15.61	0.93 ± 0.54	2.35 ± 2.08	0.81 ± 0.26
Glucose [mg/dL]	70–110	96.77 ± 10.27	89.62 ± 12.1	92.67 ± 8.5	91.75 ± 5.97
Creatinine [mg/dL]	0.50–0.90	0.88 ± 0.16	0.82 ± 0.12	0.79 ± 0.08	0.76 ± 0.12
ALT [U/I]	0–31	21.10 ± 19.17	24.17 ± 17.20	17.75 ± 11.27	12 ± 5.24
AST [U/I]	0–32	15.59 ± 5.26	22.42 ± 9.23	17 ± 3.67	16.33 ± 3.21
CSF analysis					
Cytosis [cells/µL]	0–5	169.7 ± 115.64	26.90 ± 25.59	99 ± 120.57	14.8 ± 12.15
Protein [mg/dL	15–45	75.95 ± 20.56	54.46 ± 26.32	69 ± 58.49	48.2 ± 27.74

### Methods

#### Antioxidant status (TAS) determination

The total antioxidant status (TAS) of the blood serum of patients and healthy subjects was determined using 2,20-azino-bis-3-ethylbenzothiazolin-6-sulfonic acid (ABTS) [16]. For this purpose, a solution ABTS cationic radical was prepared in phosphate buffer (PBS, pH 7.4). 3 times diluted blood serum samples (5 μL) were incubated (37 °C) with ABTS radical working solution (245 μL) for 10 min. Absorbance was measured using an Infinite 200 plate reader (TEKAN, Switzerland) at 734 nm.

The test results are reported with reference to the equivalent antioxidant capacity of Trolox (TEAC) as a standard. Trolox (Hoffman-LaRoche, Basel, Switzerland) is a water-soluble derivative of vitamin E with antioxidant properties and does not interact with other cellular components. The TAS level is therefore expressed in mmol Trolox/l.

#### Phospholipid metabolism estimation

##### Free and phospholipid fatty acids level

The phospholipid and free fatty acids were analyzed by gas chromatography [17]. Fatty acids were isolated by Folch extraction using chloroform/methanol mixture (2:1, v/v) in the presence of 0.01% butylated hydroxytoluene. Using TLC analytes were separated with the mobile phase as follows: heptane-diisopropyl ether–acetic acid (60:40:3, v/v/v). All lipid fractions were transmetylated to fatty acid methyl esters (FAMEs) with boron trifluoride in methanol. FAMEs were analyzed by gas chromatography with a flame ionization detector (FID) on Clarus 500 Gas Chromatograph (Perkin Elmer). Separation of FAMEs was carried out on capillary column coated with Varian CP-Sil88 stationary phase (50 m × 0.25 mm, ID 0.2 μm, Varian). Identification of FAMEs was made by comparison of their retention time with standards and quantitation was achieved using an internal standard method (nonadecanoic acid (19:0) and 1,2-dinonadecanoyl-sn-glycero-3-phosphocholine (19:0 PC) were used as internal standards). Plasma levels of PL-AA, PL-DHA, free AA, and free DHA, were expressed in μg/mL.

##### Lipid peroxidation products level

Lipid peroxidation in plasma was estimated by measuring small molecular weight reactive aldehyde, malondialdehyde (MDA) as well as neuroprostanes (NPs). The reactive aldehyde was determined using gas chromatography coupled with mass spectrometry 7890A GC–7000 (Agilent Technologies, Palo Alto, CA, USA) as the O-pentafluorobenzyl-oxime (O-PFB-oxime) or O-pentafluorobenzyl-oxime-trimethyl silane (O-PFB-oxime-TMS) derivatives, based on Luo’s method [18]. Benzaldehyde-d6 was added to plasma as an internal standard. Aldehyde derivatives were separated using an HP- 5 ms capillary column (0.25-mm internal diameter, 0.25-μm film thickness, 30-m length) and analyzed in selected ion monitoring mode (SIM). Samples were deproteinized by the addition of 1 mL of methanol. MDA derivatives were extracted with hexane. The hexane layer was evaporated and N,o-bis (trimethylsilyl) trifluoroacetamide in 1% trimethylchlorosilane was added. The following ions were monitored: m/z 204.0 and 178.0 for MDA-PFB and m/z 307.0 for IS (benzaldehyde-D6) derivatives. Plasma level of MDA was expressed in nmol/mL.Total NPs were quantified using modified LC–MS methods of Coolen and Fam respectively [19]. NPs were isolated using SPE method, after an alkaline hydrolysis step. All analyses were performed using an Agilent 1290 UPLC system interfaced with an Agilent 6460 triple quadrupole mass spectrometer with electrospray ionization source (ESI). The separation was performed using a reverse phase C18 column and linear gradient water (pH 5.7) and acetonitrile. NPs were analyzed by selected ion monitoring (SIM) in the m/z 357, as a series of peaks that have molecular masses and retention times expected for NPs generated from the oxidation of DHA in vitro.

##### PLA2, COX1/2 and LOX5 activity

The activity of enzymes involved in the metabolism of phospholipids and fatty acids was examined spectrophotometrically using commercially available assay kits, phospholipase A2 (PLA2–EC.3.1.1.4; Cayman Chemical Company, Ann Arbor, MI, USA), cyclooxygenases 1 and 2 (COX-1/2–EC.1.14.99.1; Cayman Chemical Company, Ann Arbor, MI, USA), and lipoxygenase-5 (LOX; Sigma-Aldrich, St. Louis, MO, USA), following the manufacturer’s instructions. The samples were incubated with arachidonoyl thiophosphatidylcholine, a synthetic substrate of cPLA2. The hydrolysis of arachidonoylthio-phosphatidylcholine by PLA2 released the free thiol, which was converted to NTB via a reaction with DTNB. The NTB concentration was determined with a spectrophotometric analysis at 405 nm.

PLA2 activity was expressed in nmol of arachidonoyl thio-phosphatidylcholine/min/mL Cayman’s COX colorimetric inhibitor screening assay measures the peroxidase components of COXs. The peroxidase activity was assessed colorimetrically by monitoring the appearance of oxidized N,N,N′- and n′-tetramethyl-phenylenediamine (TMPD) at 590 nm. One unit of COX’s enzyme activity is defined as the amount of enzyme that will oxidize 1.0 nmol of TMPD per minute at 25 ℃. The specific COX1-inhibitor SC-560 was applied to measure only COX-2 activity. Cyclooxygenases’ activities were expressed in U/mL.

In the Sigma-Aldrich lipoxygenase-5 (LOX-5) activity assay, lipoxygenase converts the LOX substrate to an intermediate that reacts with the probe generating a fluorescent product. The increase in the fluorescent signal can be recorded at Ex/Em 500/536 nm and is directly proportional to LOX-5 activity. One unit (U) of LOX-5 was determined as the amount of enzyme that causes oxidation of 1 μmol of the LOX probe per minute at pH 7.4 and at room temperature. LOX-5 activity was expressed in U/mL.

##### Endocannabinoids level

An analysis of endocannabinoids [anandamide (AEA) and 2-arachidonylglycerol (2-AG)] and related compounds [palmitoylethanolamide (PEA)] was performed using a Shimadzu UPLC system (Nexera X2) coupled with an electrospray ionization source (ESI) to a Shimadzu 8060 Triple Quadrupole system (Shimadzu, Kyoto, Japan) operating in the positive ion mode [20]. Analyte separation was performed using a 120 EC-C18 analytical column (3.0 × 150 mm; a 2.7 μm particle size) with a 5 μL injection volume. The mobile phase consisted of (A) 0.1% formic acid in MilliQ water and (B) 0.1% formic acid in acetonitrile. The following gradient was employed: 0.0–5.0 min, 70–80% B; 5.0–10.0 min, 80–88% B; 10.0–16.0 min, 78–100% B; 16.0–20.0 min, 100% B; 20.0–21.0 min, 100–70% B; and 21.0–25.0 min, 70% B. Briefly, cell culture samples were thawed on ice and spiked with 10 μL of an internal standard solution (100 ng/mL of AEA-d8, 2-AG-d8, and OEA-d4) and then applied to pre-washed and conditioned solid phase extraction SPE cartridges. After loading the sample, the cartridges were washed, dried under a high vacuum, and eluted. Eluates were concentrated and reconstituted in 30 μL of ACN/H2O (7:3) with 0.1% formic acid and vortexed (if necessary, they were centrifuged to remove any residuals). Solutions were then transferred to LC vials with low-volume inserts and an LC–MS/MS analysis was performed immediately. Transitions of the precursors to the product ions were as follows: m/z 348.3 → 62.15 for AEA, m/z 379.3 → 287.25 for 2-AG, m/z 300.3 → 62.0 for PEA, m/z 356.2 → 63.05 for AEA-d8, m/z 387.3 → 294.0 for 2-AG-d8, and m/z 330.20 → 66.15 for OEA-d4. The level of endocannabinoids was determined using a calibration curve range of 1–100 pmol/mL for AEA (r^2^−0.9992); of 10–1000 pmol/mL for 2-AG (r^2^−0.9995); of 10–500 pmol/mL for PEA (r^2^−0.9993). The levels of endocannabinoids were expressed in pmol/mL.

##### Eicosanoids level

Eicosanoids analysis was performed using an Shimadzu UPLC system (Nexera X2) coupled with an electrospray ionization source (ESI) to a Shimadzu 8060 Triple Quadrupole mass spectrometer (Shimadzu, Kyoto, Japan) operating in negative mode [21]. Analyte separation was performed using an Eclipse Plus C18 analytical column (2.1 × 100 mm, 1.8 µm particle size) with 3 µL injection volume. The mobile phase consisted of 0.1% acetic acid in MilliQ water (A) and acetonitrile (B). The following gradient was employed: 0.0–1.0 min 25–40% B, 1.0–2.5 min 40–42% B, 2.5–4.5 min 42–50% B, 4.5–10.5 min 50–65% B; 10.5–12.5 min 65–75% B; 12.5–14.0 min 75–85% B; 14.0–14.5 min 85–95% B; 14.5–15 min 95–25% B; 15.0–16.0 min 25% B. Briefly, plasma samples were thawed on ice and spiked with 10 µL internal standard solution (100 ng/mL TXB2-d4, PGD2-d4, 15-d-PGJ2-d4 and 15-HETE-d8) and then applied to pre-washed and conditioned solid phase extraction SPE cartridges. After loading the sample the cartridges were washed, dried under high vacuum and eluted. Eluates were concentrated and reconstituted in ACN/H2O (8:2) with 0.1% acetic acid and vortexed (if necessary centrifuged to remove any residuals). Solutions were then transferred to LC vials with low-volume inserts and LC–MS/MS analysis was performed immediately. The precursor to the product ion transitions were as follows: m/z 351.3 → 271.2 for PGE2, m/z 315.2 → 271.2 for 15-d-PGJ2, m/z 369.3 → 169.1 for TXB2, m/z 355.0 → 275.3 for PGD2-d4, m/z 373.0 → 173.1 for TXB2-d4 m/z 319.3 → 275.2 for 15-d-PGJ2-d4. Levels of eicosanoids were expressed as pmol/mL.

#### Protein expression determination

Receptor expression was measured using an enzyme-linked immunosorbent assay (ELISA) [22] Cell lysates (granulocytes and lymphocytes) were applied to the wells of an ELISA plate (Nunc Immuno MaxiSorp, Thermo Scientific, Waltham, MA, USA). The plates with attached proteins were incubated at 4 °C for 3 h with blocking solution (5% skimmed milk powder in carbonate binding buffer). After washing with PBS supplemented with 0.1% Tween 20, samples were incubated at 4 °C overnight with appropriate primary antibodies against TRPV1 (host: mouse) (Sigma-Aldrich, St. Louis, MO, USA); CB1, CB2, (host: mouse) (Santa Cruz Biotechnology, CA, USA); PPARγ (host: rabbit) (Invitrogen, Waltham, MA, USA). All antibodies were used at a concentration of 1:1000. Then, after washing (PBS supplemented with 0.1% Tween 20), the plates were incubated for 30 min with peroxidase blocking solution (3% H2O2, 3% skimmed milk powder in PBS) at room temperature. Goat anti-rabbit/mouse antibody EnVision + Dual Link/HRP solution (1:100) (Agilent Technologies, Santa Clara, California, USA) was used as secondary antibody. After 1 h of incubation at room temperature, secondary antibodies were removed and plates were incubated with chromogen substrate solution (0.1 mg/mL TMB, 0.012% H_2_O_2_) for 40 min. The reaction was stopped by adding 2 M sulfuric acid and the absorbance was read after 10 min at 450 nm and automatically recalculated from standard curves for each protein CB1: Abcam, Cambridge, UK; CB2: Abnova, Taipei, Taiwan; TRPV1: Lifespan Biosciences, Seattle, WA, USA; PPARγ: Fine, Wuhan Test, Hubei, China;). The values obtained were converted to mg of protein in the samples.

#### Statistical analysis

Data were expressed as mean ± SD, and were analyzed by one-way analysis of variance (ANOVA) followed by a post hoc Tukey testing using Statistica software (Statistica 13.3, StatSoft, Poland). Results were compared using Mann–Whitney U test and Wilcoxon signed-rank test. Values of p ≤ 0.05 were considered significant, and only these results were discussed in detail.

## Results

**Fig. 1:**
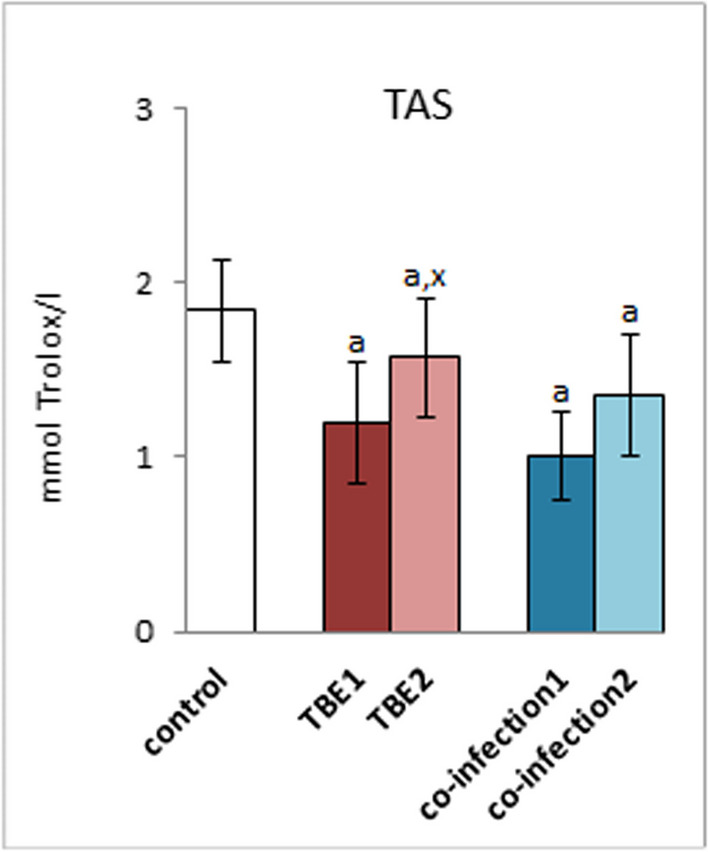
The total antioxidant status (TAS) in plasma of patients with TBEV infection (TBE1—before therapy and TBE2—after therapy) and patients with TBEV + Bb/Ap co-infection (co-infection1—before therapy and co-infection2—after therapy) was compared to healthy subjects (control). The differences between mean values was indicated for p < 0.05: TBE patients (n = 40) before and after treatment (TBE1 or TBE2) or patients with TBEV + Bb/Ap co-infection (n = 6) before and after treatment (co-infection1 or co-infection2) and control group (n = 20) was signed as “a”. TBE patients (n = 40) after (TBE2) and before (TBE1) treatment (n = 40) was signed as “x”

The results of this study showed that infection with TBEV as well as co-infections with the virus and bacteria (TBEV + Bb/Ap) causes a decrease in the total antioxidant status (TAS) in the blood plasma of patients compared to the values in the group of healthy people (control group) (Fig. 1). However, no statistically significant differences were observed in the TAS values, which were assessed before treatment, in both groups of patients (TBE1 and coinfections1) before treatment. Moreover, it was found that as a result of therapy in patients with TBE (TBE2), there was a significant increase in TAS values, but these values did not reach the values in the group of healthy people (control). Additionally, in the plasma of treated patients with co-infection (co-infection2), no increase in TAS levels was observed.

**Fig. 2:**
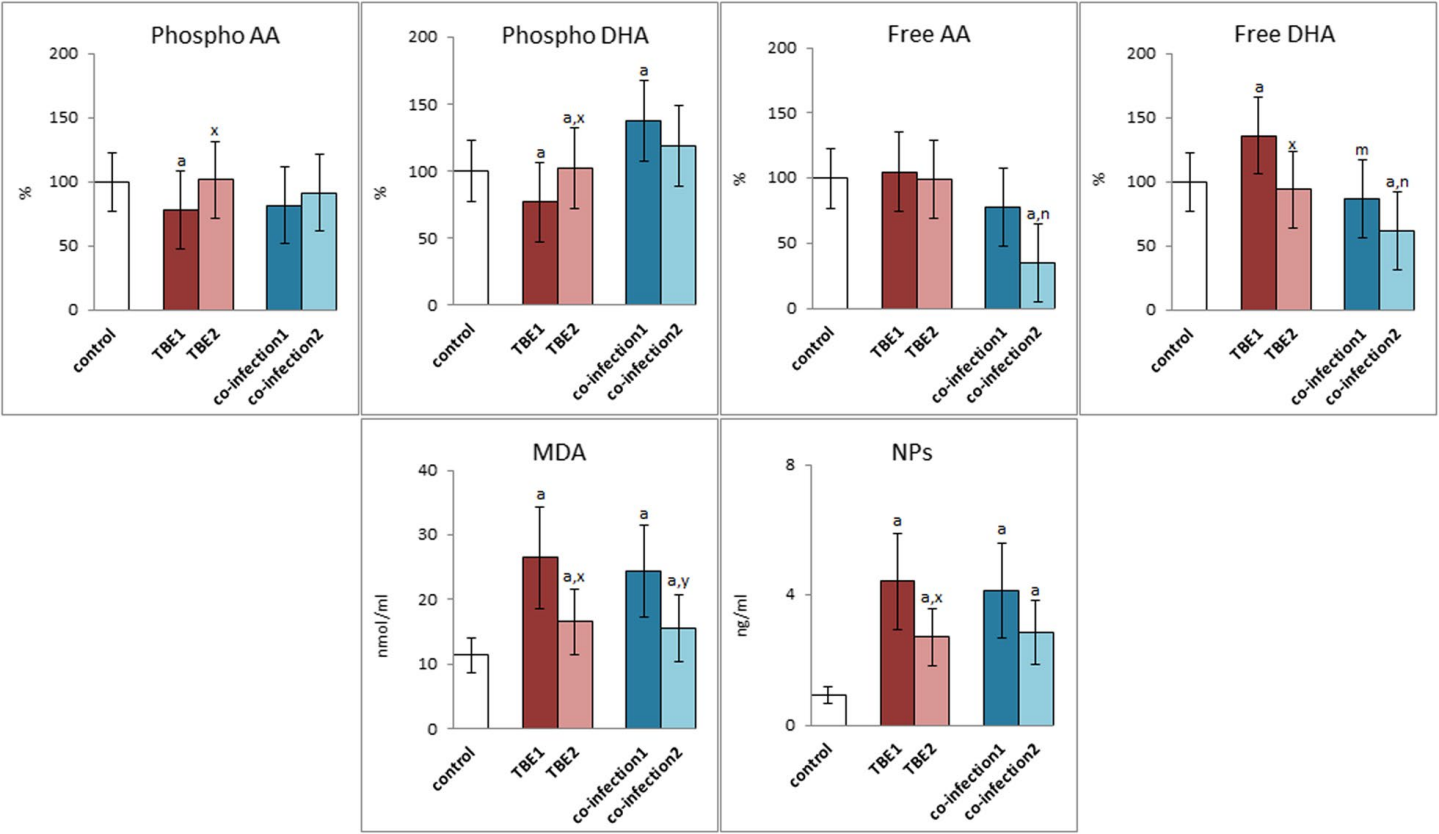
The level of phospholipid (Phospho)and free polyunsaturated fatty acids (Free) (arachidonic acid (AA) and docosahexaenoic acid (DHA)) as well as the expression of lipid peroxidation products (malonyldialdehyde (MDA) and neuroprostanes (NPs) in plasma of patients with TBEV infection (TBE1—before therapy and TBE2—after therapy) and patients with TBEV + Bb/Ap co-infection (co-infection1—before therapy and co-infection2—after therapy) was compared to healthy subjects (control). The differences between mean values was indicated for p < 0.05: TBE patients (n = 40) before and after treatment (TBE1 or TBE2) or patients with TBEV + Bb/Ap co-infection (n = 6) before and after treatment (co-infection1 or co-infection2) and control group (n = 20) was signed as “a”. TBE patients (n = 40) after (TBE2) and before (TBE1) treatment (n = 40) was signed as “x”. TBEV + Bb/Ap coinfected patients (n = 6) after (co-infection2) and before (co-infection1) treatment was signed as “y”. TBEV + Bb/Ap coinfected patients (n = 6) before treatment (co-infection1) and TBE patients (n = 40) before treatment (TBE1) was signed as “m”. TBEV + Bb/Ap coinfected patients (n = 6) after treatment (co-infection2) and TBE patients (n = 40) after treatment (TBE2) g was signed as “n”

The decrease of antioxidant capacity observed in the patients' blood plasma favored the intensification of oxidative reactions, including those related to phospholipids and free polyunsaturated fatty acids (PUFAs) (Fig. 2). The level of phospholipid docosahexaenoic acid (DHA) and arachidonic acid (AA) in the plasma of patients with TBE was significantly reduced before treatment but the treatment of patients resulted in the return of both examined PUFAs level to the level observed in the plasma of healthy people (control). In the case of bacterial co-infections, the level of phospholipid PUFAs (AA and DHA) before treatment was decreased (AA) and increased (DHA), while after treatment the values ​​tended to normalize (to reach control values). However, due to the small size of these groups, these changes were not statistically significant. However, in the case of free PUFAs, TBE infection promoted a significant increase in DHA levels, while AA levels were not changed. The therapy resulted in the return of free DHA levels to control values. In the case of co-infections, the levels of free AA and DHA before treatment were at the control level, and only after treatment they were significantly reduced.

Since changes in PUFAs level may be a consequence of their altered metabolism dependent on both ROS and lipolytic enzymes, the level of products from oxidative fragmentation and cyclization of PUFAs was assessed. In the plasma of TBE and co-infected patients (TBE1 and co-infection1), there was a significant increase in the level of the product of PUFAs oxidative fragmentation—malondialdehyde (MDA) and the product of oxidative cyclization—neuroprostanes (NPs) compared to the concentration in the plasma of healthy people (Fig. 2). However, treatment of patients with infection (TBE2) and co-infection (co-infection2) led to a statistically significant reduction in the level of both lipid peroxidation products in the group of TBE patients, which was still higher than in the control group. This especially concerned the level of neuroprostanes.

**Fig. 3:**
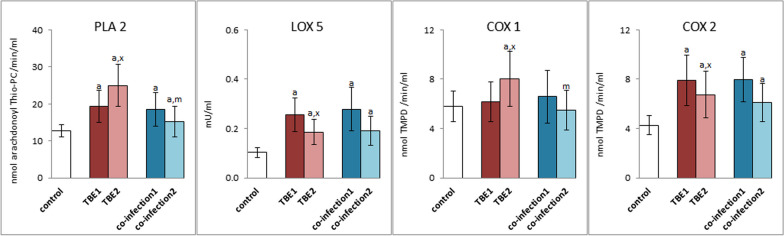
The expression of phospholipase A2 (PLA 2), lipoxygenases (LOX 5) and cyclooxygenases (COX 1 and 2) in plasma of patients with TBEV infection (TBE1—before therapy and TBE2—after therapy) and patients with TBEV + Bb/Ap co-infection (co-infection1—before therapy and co-infection2—after therapy) was compared to healthy subjects (control). The differences between mean values was indicated for p < 0.05: TBE patients (n = 40) before and after treatment (TBE1 or TBE2) or patients with TBEV + Bb/Ap co-infection (n = 6) before and after treatment (co-infection1 or co-infection2) and control group (n = 20) was signed as “a”. TBE patients (n = 40) after (TBE2) and before (TBE1) treatment (n = 40) was signed as “x”. TBEV + Bb/Ap coinfected patients (n = 6) before treatment (co-infection1) and TBE patients (n = 40) before treatment (TBE1) was signed as “m”

The conducted research allowed to conclude that oxidative stress also causes an increase in the activity of enzymes responsible for the metabolism of phospholipids (PLA2) and free PUFAs (COXs and LOX). Both TBE and co-infections have been shown to cause statistically significant increases in the activity of phospholipase A2 (PLA2), lipoxygenase 5 (LOX5) and the inducible cyclooxygenase isoenzyme (COX2) (Fig. 3). However, the therapy used in the case of TBE (KZM2) resulted in an increase in the activity of PLA2 and COX1 and a decrease in the activity of other enzymes. In the case of coinfection, the therapy resulted in a decrease in the activity of all analyzed enzymes.

**Fig. 4:**
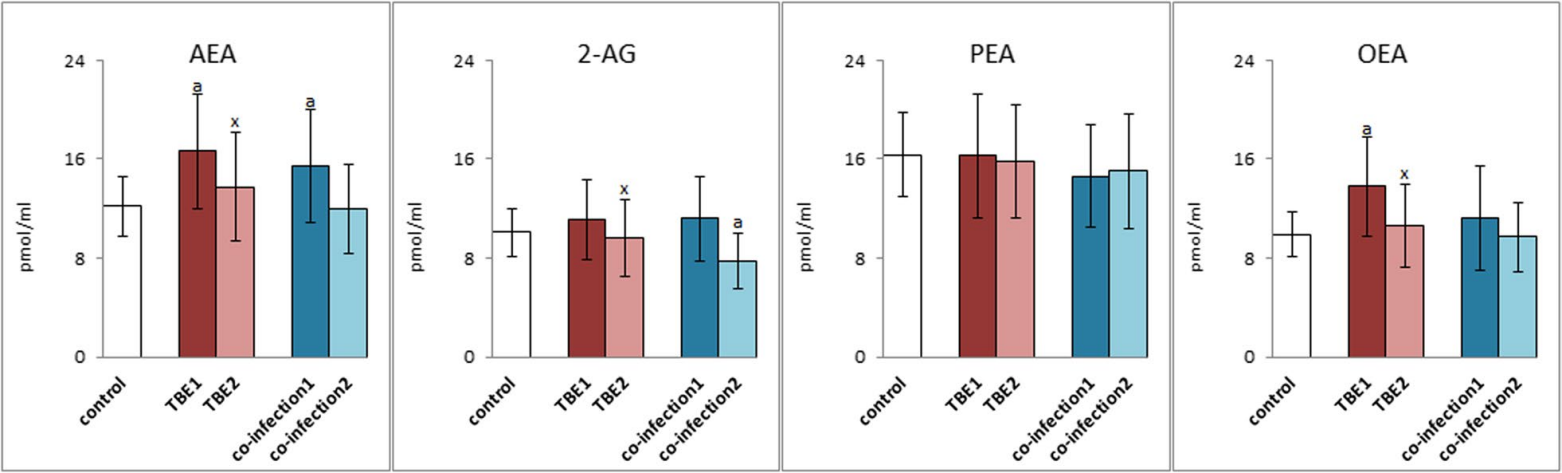
The expression of anandamide (AEA), 2-arachidonoylglycerol (2-AG), palmitoylethanolamine (PEA) and oleoylethanolamide (OEA) in plasma of patients with TBEV infection (TBE1—before therapy and TBE2—after therapy) and patients with TBEV + Bb/Ap co-infection (co-infection1—before therapy and co-infection2—after therapy) was compared to healthy subjects (control). The differences between mean values was indicated for p < 0.05: TBE patients (n = 40) before and after treatment (TBE1 or TBE2) or patients with TBEV + Bb/Ap co-infection (n = 6) before and after treatment (co-infection1 or co-infection2) and control group (n = 20) was signed as “a”. TBE patients (n = 40) after (TBE2) and before (TBE1) treatment (n = 40) was signed as “x”

Changes in the level of phospholipid PUFAs are directly related to changes in the level of lipid mediators, both from the group of endocannabinoids and eicosanoids. TBEV infection caused a significant increase in the levels of anandamide (AEA) and oleoylethanolamide (OEA), the levels of which were significantly reduced after pharmacotherapy to values close to those in the control group (Fig. 4). In the case of bacterial co-infections (TBE + LB/HGA), this direction of change concerned only AEA. However, in the plasma of patients with bacterial co-infections, the level of 2-AG was significantly reduced after treatment.

**Fig. 5:**
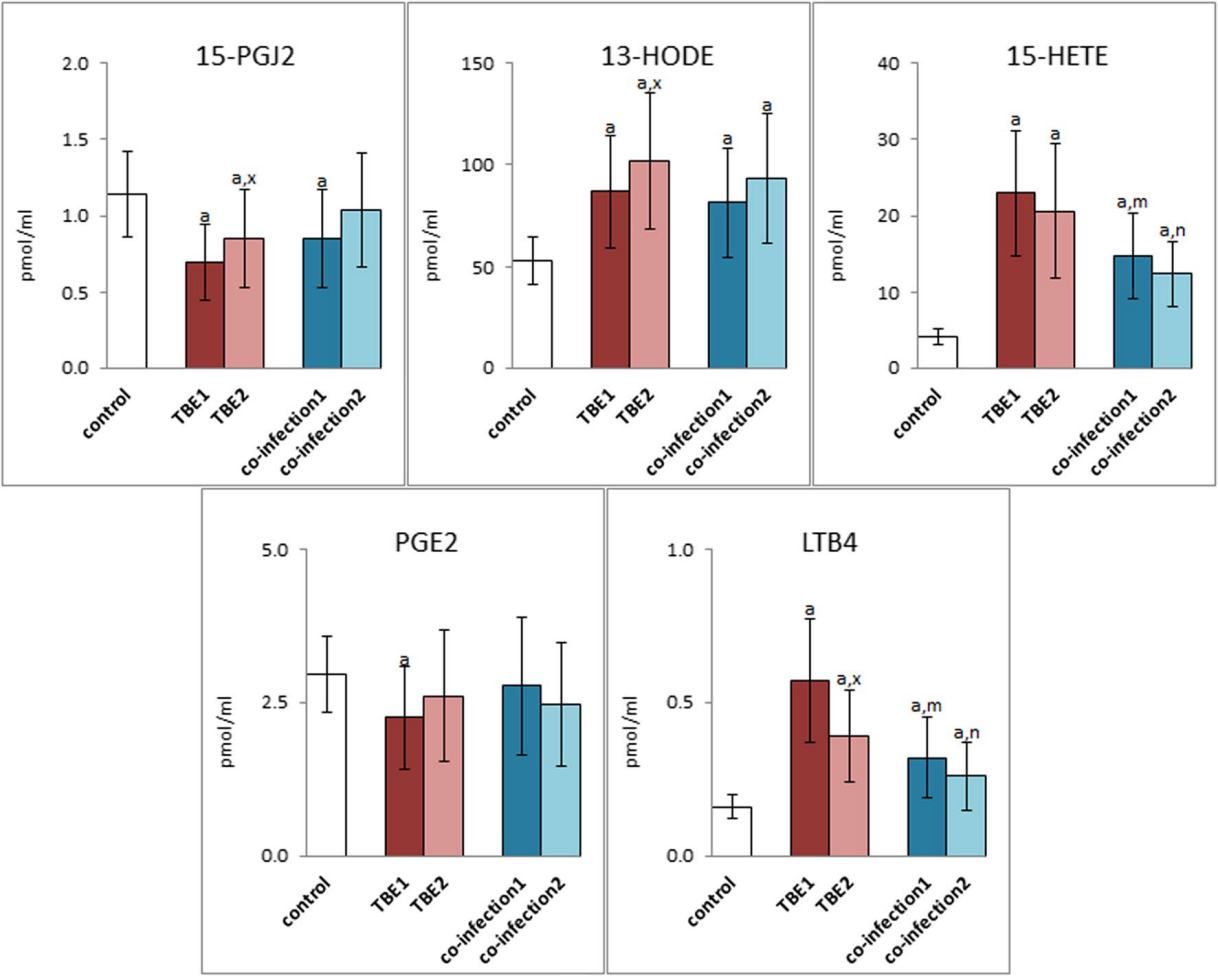
The expression of anti-inflammatory eicosanoids such as 15-prostaglandin J2 (15-PGJ2), 13-hydroxyoctadecadienoic acid (13-HODE), 15-hydroxyeicosatetraenoic acid (15-HETE), as well as pro-inflammatory prostaglandin E2 (PGE2) and leukotriene B4 (LTB4) in plasma of patients with TBEV infection (TBE1—before therapy and TBE2—after therapy) and patients with TBEV + Bb/Ap co-infection (co-infection1—before therapy and co-infection2—after therapy) was compared to healthy subjects (control). The differences between mean values was indicated for p < 0.05: TBE patients (n = 40) before and after treatment (TBE1 or TBE2) or patients with TBEV + Bb/Ap co-infection (n = 6) before and after treatment (co-infection1 or co-infection2) and control group (n = 20) was signed as “a”. TBE patients (n = 40) after (TBE2) and before (TBE1) treatment (n = 40) was signed as “x”. TBEV + Bb/Ap coinfected patients (n = 6) before treatment (co-infection1) and TBE patients (n = 40) before treatment (TBE1) was signed as “m”. TBEV + Bb/Ap coinfected patients (n = 6) after treatment (co-infection2) and TBE patients (n = 40) after treatment (TBE2) g was signed as “n”

However, changes in the level of free PUFAs in the plasma of patients are also accompanied by changes in the level of eicosanoids with anti-inflammatory (13-HODE, 15-HETE, 15-PGJ2) and pro-inflammatory (LTB4, PGE2) effects (Fig. 5). Increased levels of 13-HODE and 15-HETE and statistically reduced levels of 15-PGJ2 were found in the plasma of patients with TBE before treatment. After therapy, the level of 13-HODE was further increased, and the level of 15-PGJ2 also increased, but did not reach the value in the control group. The direction of changes in the above eicosanoids in the plasma of patients with bacterial co-infections was the same as in the plasma of TBE patients. However, the level of pro-inflammatory LTB4 was several-fold increased in TBE patients before treatment and was significantly decreased after treatment. A similar direction of changes was observed in patients with co-infections. On the other hand, the level of prostaglandin PGE2 in patients with TBE was decreased before treatment and increased after treatment, while no significant changes in the level of this prostaglandin were observed in the patients' plasma.

**Fig. 6:**
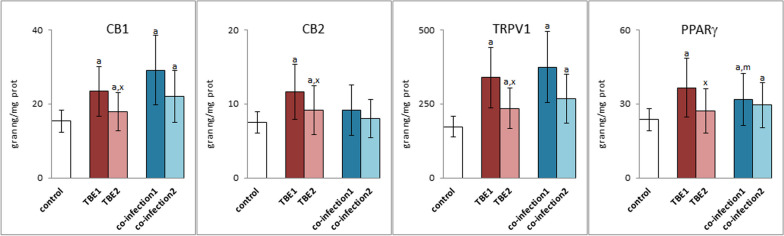
The expression of membrane receptors: CB1, CB2, TRPV1 and PPARγ in granulocytes of patients with TBEV infection (TBE1—before therapy and TBE2—after therapy) and patients with TBEV + Bb/Ap co-infection (co-infection1—before therapy and co-infection2—after therapy) was compared to healthy subjects (control). The differences between mean values was indicated for p < 0.05: TBE patients (n = 40) before and after treatment (TBE1 or TBE2) or patients with TBEV + Bb/Ap co-infection (n = 6) before and after treatment (co-infection1 or co-infection2) and control group (n = 20) was signed as “a”. TBE patients (n = 40) after (TBE2) and before (TBE1) treatment (n = 40) was signed as “x”. TBEV + Bb/Ap coinfected patients (n = 6) before treatment (co-infection1) and TBE patients (n = 40) before treatment (TBE1) was signed as “m”

Changes in the level of endocannabinoids and eicosanoids resulted in the increased expression of CB1, CB2, TRPV1 and PPARγ receptors in granulocytes of TBE patients (Fig. 6). However, in the case of bacterial co-infections, only increased expression of CB1, TRPV1 and PPARγ is observed. Relief of disease symptoms, especially in TBE, resulted in a significant reduction in the expression of all labeled receptors. However, only in the case of CB1/2 and TRPV1 receptors, these values differed statistically significantly from the values before treatment. In the case of bacterial co-infections, drug therapy also contributed to reducing the expression level of the tested receptors, but without statistical significance. Apart from the CB2 level, all other granulocyte receptors in patients with co-infections (TBE + LB/HGA) and after pharmacotherapy are characterized by higher expression than in granulocytes of the control group.

## Discussion

One of the basic conditions of the human body physiological state is the redox balance observed both at the level of cells and tissues, including body fluids such as blood. This is due to the fact that the physiological generation of ROS is balanced by effective enzymatic and non-enzymatic antioxidants [23]. This is conducive to the proper functioning of the human body, which requires strict control of metabolic processes, including those related to the transmission of signals from the environment, e.g. by pathogens. One of the basic elements of the humans body response to the invasion of pathogens is the found increased activity of pro-oxidative enzymes in leukocytes, which play a key role in the immune defense of the host against pathogens [24]. This response is confirmed by previous studies of blood plasma of patients with both TBEV infection and co-infections with bacteria such as *B. burgdorferi *s.l. and *A. phagocytophilum* [4]. Enhanced generation of ROS is also conducive to modulation of the signal transduction cascade and strengthening the immune function of lymphocytes in the patient's body [25]. Infection with pathogens is usually accompanied by changes in the effectiveness of endogenous antioxidant mechanisms that are responsible for maintaining the redox balance in physiological conditions [4]. This situation is consistent with the significantly reduced plasma total antioxidant status (TAS) of patients with TBEV infection and bacterial co-infections observed in this study. It is worth emphasizing that after pharmacotherapy the plasma TAS level in patients with TBE and co-infections increases, but still remains statistically significantly lower (in both groups of patients) compared to the values ​​in healthy people. This clearly indicates that standard therapy in the case of TBE, and especially bacterial co-infections, is not able to restore the patient's organism into a physiological state.

The results of previous [4] as well as this study indicate that both TBE alone and together with bacterial co-infections promote the formation of oxidative stress in the patient's body, and a similar situation applies to many diseases, including those caused by both viruses and bacteria [1]. Although oxidative stress plays a dual role in infectious diseases, ROS modify the susceptibility of the host organism to infection, as well as ROS become harmful to both the pathogen and host [26], which promotes oxidative modifications of basic components of the human body, including crucial for the host's immune response lipids and proteins [27].

In tick-borne encephalitis, the pathogen interferes both at the level of the central nervous system (CNS) and at the systemic level [28]. Because the membranes of nerve cells are exceptionally rich in phospholipids containing large amounts of PUFAs, including arachidonic and docosahexaenoic acids, which, due to the large number of unsaturated bonds, are extremely susceptible to ROS action. As a result, reactive aldehydes, including MDA, are formed, which are products of oxidative fragmentation phospholipid arachidonic acid, as well as prostaglandin derivatives—products of oxidative cyclization of hydrocarbon chains of fatty acids [29]. Due to the large amount of docosahexaenoic acid in neurons and its greater susceptibility to oxidation than arachidonic acid, neuroprostanes are formed—cyclization products of this acid (DHA), whose level changes can also be observed in the blood. Literature data indicate that in patients with TBE as a result of CNS infection, the blood–brain barrier is damaged [30], which, combined with generalized increased oxidative stress, promotes lipid peroxidation, which may explain their approximately fourfold increase of neuroprostanes in plasma concentration, and at the same time, a significant decrease in their level after pharmacotherapy. Similar situation was observed for isoprostanes [4]. In turn, the increase in the level of MDA is mainly associated with the oxidative modification of arachidonic acid, the level of which in the plasma of patients is significantly reduced. Thus, changes in the level of fatty acids and lipid peroxidation products confirm generalized oxidative stress in the body of patients with TBE and bacterial co-infections. Statistically significant decrease in the level of MDA and neuroprostanes in patients with TBE after pharmacotherapy indicates an improvement in the systemic metabolism of phospholipids and free PUFAs in this group of patients. However, in the case of TBE with bacterial co-infections, a significant reduction in the level is observed only in the case of MDA. Therefore, it can be suggested that pro-oxidative conditions still prevail in the patient's body and/or the state of the blood–brain barrier has not completely improved.

The metabolism of phospholipids is modified not only as a result of the direct reaction of PUFAs with ROS, but is also controlled by enzymes metabolizing phospholipids and free PUFAs, the activity of which is increased under conditions of oxidative stress [31]. Therefore, in states of viral infection (TBE) and bacterial co-infection (TBE + LB/HGA), PLA2 is activated, which promotes the release of both fatty acids and their cyclization products from lipid structures. There is also increased activity of COX1/2 and lipoxygenase, including LOX5, which metabolize free PUFA into lipid mediators [32]. This situation favors reducing the level of fatty acids (AA and DHA). However, previous reports have shown that in HCV infection PUFAs, mainly AA, may enhance the antiviral effect of IFN-α and contribute to the antipathogenic effect [33]. However, in TBE infection, the level of free AA does not change, while it decreases in co-infection, especially after treatment, which may be due to increased lipid peroxidation. On the other hand, a decrease in the level of AA after the end of therapy may suggest the depletion of antiviral resources in the patients' organism. On the other hand, the increase in free DHA in the blood plasma of patients with TBE may indicate the accumulation of this acid coming from both CNS and blood levels. Literature data also show that the inducible COX2 isoform is upregulated during influenza virus infection, contributing to the excessive cytokine response observed in severe H5N1 infections [34]. As a consequence, it can be suggested that a significant increase in the activity of enzymes metabolizing PUFAs may be an offensive reaction of the body to infection.

Another consequence of the increased activity of enzymes responsible for PUFA metabolism is an increased level of some lipid mediators, including endocannabinoids and eicosanoids, whose metabolic effects are mainly the result of activation of membrane receptors of blood cells [35, 36]. Among the endocannabinoids presented in this paper, AEA and OEA are characterized by a significant increase in plasma levels as a result of infection, but only AEA as a result of co-infection. It can be suggested that the increased level of endocannabinoids is mainly a consequence of the inhibition of the activity of enzymes degrading these lipid mediators in conditions of infection [37]. Literature data also indicate that in viral infections caused by HIV or Theiler virus, AEA may participate in the regulation of inflammation by lowering the level of pro-inflammatory cytokines and activating anti-inflammatory pathways in immunosuppressive cells [38]. OEA also has anti-inflammatory and antioxidant properties, which prevents damage to the vascular endothelium [39].

The consequence of increased activity of COXs and LOXs as a result of infection is increased enzymatic metabolism of free PUFAs to other lipid mediators, such as eicosanoids, which may reveal both pro- and anti-inflammatory effects. Literature data clearly indicate that viral infection increases generation of 15-HETE and 13-HODE, which belong to the pathways that modulate inflammation [35, 40]. It is known that eicosanoids act mainly through membrane receptors and that both 13-HODE and 15-HETE are agonists of PPARγ receptors, thus promoting the resolution phase of inflammation [35] and in addition, 13-HODE is also a ligand that activates the TRPV1 receptor [40], whose increased activation is observed in granulocytes of patients with both TBE and bacterial co-infections. Thus, the increase in 13-HODE concentration observed after pharmacotherapy of patients with TBE and bacterial co-infections (TBE + LB/HGA) may confirm the resolution of the disease process. In addition, 13-HODE inhibits platelet adhesion and macrophage apoptosis [35], and is also a ligand that activates the TRPV1 receptor [40], whose increased activation is observed in the granulocytes of patients with both TBE and bacterial co-infections.

However, the level of 15-HETE does not show a tendency to increase, which may be due to the fact that 15-HETE is further metabolized as a precursor of the biosynthesis of lipoxin LXB4, which is an important agonist of inflammation resolution [41]. Thus, such a change in the level of these mediators may indicate a tendency to resolution of inflammation. The results of this study indicate the same direction of changes in PLA2 and 15-HETE activity, which may also confirm their mutual interaction in the case of TBE. However, the reduced level of anti-inflammatory 15-PGJ2 observed in the plasma of patients with both TBE and bacterial co-infections, which reduces the activation of the pro-inflammatory transcription factor NFκB [42], indicates the presence of inflammation in patients with both TBE and co-infections.

Moreover, in the early stages of TBE and viral-bacterial co-infections, elevated levels of leukotriene B4 (derived from AA) are observed in the plasma of patients, which are involved in the early stages of inflammation during infection [43]. It is known that LTB4 promotes the migration and survival of neutrophils, increases the secretion of enzymes from neutrophil granules and the production of superoxide anion radicals [44]. These leukotrienes may also participate in the recruitment of T lymphocytes to sites of inflammation [45]. In addition, LTB4 has been shown to induce the release of an antimicrobial peptide from neutrophils and, in some cases, inhibit viral replication [44]. It can therefore be suggested that the several-fold increase in LTB4 observed at the onset of TBEV infection stimulates the antiviral response against pathogens. On the other hand, in viral-bacterial co-infection, there is a much smaller increase in LTB4 levels and a smaller reduction after treatment. However, studies on the metabolic consequences of *Mycobacterium tuberculosis* infection have shown that the correct LTB4/PGE2 ratio is essential in controlling the host immune response, suggesting that optimal PGE2 production is also required for an effective immune response [46]. This may suggest that the situation may be similar for other infections, including bacterial co-infections. Thus, in the case of bacterial co-infections, no changes in the level of this prostaglandin are observed, while in the plasma of patients with TBE, reduced levels of PGE2 are observed before treatment, which may indicate a weakened immune response.

It is known, however, that in addition to the role of modulators in the central nervous system, endocannabinoids as well as eicosanoids are also peripheral immune mediators, with anti-inflammatory or pro-inflammatory effects depending on the representative and the receptors activated by it. These lipid mediators are known to act as agonists of the G protein-bound receptors, including CB1, CB2, TRPV1 and PPARγ receptors, the increased expression of which is observed in the present study in the granulocytes of patients with TBE and bacterial co-infection. However G protein-bound receptors are involved in the regulation of redox balance and inflammation [47] and activation of CB1 receptors increases the generation of ROS and the pro-inflammatory factor TNF-α, while activation of CB2 receptors promotes the reduction of ROS and TNF-α generation [48], which may contribute to the reduction of the pro-inflammatory and stress response in viral and bacterial infection. Such a multidirectional effect is indicated by a strong activation of CB1 receptors and at the same time a much lower activation of CB2 receptors in the granulocytes of patients with co-infections (KZM + LB/HGA). In addition, CB2 receptors not only reduce oxidative stress and inflammation, but also regulate mitogen-activated protein kinases (MAPK) and indirectly stimulate phospholipases, including PLA2 [49, 50], which may indicate a feedback loop and further intensification of phospholipid metabolism, as may indicate reduced levels of free PUFAs, especially after co-infection. It is also known that by activating the PPARγ family of receptors, endocannabinoids inhibit inflammatory responses and enhance the host response to inflammation [51]. This seems to be confirmed by the results of the present study, which indicate significantly less activation of PPARγ in the case of co-infection than in the case of TBEV infection alone. In contrast to other viral diseases such as COVID-19, it has been suggested that OEA, which is also upregulated only in TBE patients, is responsible for the downregulation of NFκB-related gene expression [52] and also modulates the effects partially mediated by PPARα [53]. In addition, in patients with COVID-19, it was found that activation of the endocannabinoid system reduces viral replication and the level of pro-inflammatory cytokines [54], hence a similar response of the endocannabinoid system, especially in the case of TBE, but also partially in co-infections, can be considered beneficial and even pro- survival effect on the infected organism.

Taking into account changes in the level of lipid mediators, both from the group of endocannabinoids and eicosanoids, and the granulocyte receptors they activate, which may be activated as a result of interaction with various lipid mediators, it is not possible to clearly link and compare changes in the levels of lipid mediators and activated receptors with the course of TBE and bacterial co-infections and with clear effects of therapy.

## Conclusion

The analysis of the observed changes in the metabolism of phospholipids and free PUFAs in the plasma of patients with TBE and bacterial co-infections (TBE + LB/HGA) indicates an intensification of the metabolism of PUFAs, leading in most cases to the overproduction of their metabolites. This applies to both metabolites produced as a result of oxidative stress as well as lipid mediators produced in enzymatic reactions, which directly or through the activation of membrane receptors participate in further metabolic transformations in the human body, which makes it difficult to clearly determine the consequences of these modifications. A partial reduction of the observed changes as a result of classic pharmacotherapy only indicates the regression of the disease process, and not the body's return to its physiological state. Therefore, taking into account the presented results and the fact that the disturbed metabolism of PUFAs is directly or indirectly related to increased oxidative stress, in order to limit the consequences of persistent oxidative stress, it can be suggested to introduce substances with antioxidant effect, which should contribute to reducing the effects of infection and faster convalescence.

## Data Availability

The datasets used and analysed during the current study available from the corresponding author on reasonable request.

## Abbreviations

13-HODE: 13-S-hydroxyoctadecadienoic acid
15-HETE: 15-Hydroxyeicosatetraenoic acid
15d-PGJ2: 15-Deoxy-delta12,14-prostaglandin J2
2-AG: 2-Arachidonylglicerol
4-HNE: 4-Hydroxynonenal
AA: Arachidonic acid
ABTS: 2,2′-Azino-bis-3-ethylbenzthiazoline-6-sulfonic acid
AEA: Anandamide
ANOVA: Analysis of variance
Ap: *Anaplasma phagocytophilum*
Bb: *Borrelia burgdorferi *Sensu lato
CNS: Central nervous system
COX: Cyclooxygenase
CSF: Cerebrospinal fluid
DHA: Docosahexaenoic acid
EAN: European Academy of Neurology
EDTA: Ethylenediamine tetraacetic acid
ESI: Electrospray ionization source
FAME: Fatty acid methyl ester
FID: Flame ionization detector
HGA: Human granulocytic anaplasmosis
LB: Lyme borreliosis
LOX: Lipoxygenase
LTB4: Leukotriene B4
MAPKs: Mitogen-activated protein kinases
MDA: Malondialdehyde
MS/MS: Tandem mass spectrometry
NFκB: Nuclear factor κ of activated B cells
NP: Neuroprostanes
Nrf2: Nuclear factor erythroid 2-related factor 2
OEA: N-oleoylethanolamine
PC: Phosphocholine
PEA: N-palmitoylethanol amine
PGE2: Prostaglandin E2
PLA2: Phospholipase A2
PUFAs: Polyunsaturated fatty acids
ROS: Reactive oxygen species
S1P: Sphingosine-1-phosphate
TAS: Total antioxidant status
TBE: Tick-borne encephalitis
TBEV: Tick-borne encephalitis virus
UPLC: Ultra-performance liquid chromatography

## References

[1] Groth M, Skrzydlewska E, Dobrzyńska M, Pancewicz S, Moniuszko-Malinowska A (2022). Redox imbalance and its metabolic consequences in tick-borne diseases. Front Cell Infect Microbiol.

[2] Dunaj J, Moniuszko-Malinowska A, Swiecicka I, Andersson M, Czupryna P (2018). Tick-borne infections and co-infections in patients with non-specific symptoms in Poland. Adv Med Sci.

[3] Riccardi N, Antonello RM, Luzzati R, Zajkowska J, Di Bella S (2019). Tick-borne encephalitis in Europe: a brief update on epidemiology, diagnosis, prevention, and treatment. Eur J Internal Med.

[4] Dobrzyńska M, Moniuszko-Malinowska A, Jarocka-Karpowicz I, Czupryna P, Groth M (2022). Metabolic response to tick-borne encephalitis virus infection and bacterial co-infections. Pathogens.

[5] Kuzmenko YV, Smirnova OA, Ivanov AV, Starodubova ES, Karpov VL (2016). Nonstructural protein 1 of tick-borne encephalitis virus induces oxidative stress and activates antioxidant defense by the Nrf2/ARE pathway. Intervirology.

[6] Gęgotek A, Skrzydlewska E (2019). Biological effect of protein modifications by lipid peroxidation products. Chem Phys Lipids.

[7] Liu T, Zhang L, Joo D, Sun SC (2017). NF-κB signaling in inflammation. Signal Transduct Target Ther.

[8] Lorizate M, Kräusslich HG (2011). Role of lipids in virus replication. Cold Spring Harb Perspect Biol.

[9] Kasuga Y, Zhu B, Jang KJ, Yoo JS (2021). Innate immune sensing of coronavirus and viral evasion strategies. Exp Mol Med.

[10] Staring J, Raaben M, Brummelkamp TR (2018). Viral escape from endosomes and host detection at a glance. J Cell Sci.

[11] Czupryna P, Mroczko B, Pancewicz S, Muszynski P, Grygorczuk S (2019). Assessment of the tau protein concentration in patients with tick-borne encephalitis. Eur J Clin Microbiol Infect Dis.

[12] Czupryna P, Grygorczuk S, Pancewicz S, Świerzbińska R, Zajkowska J (2018). Evaluation of NSE and S100B in patients with tick-borne encephalitis. Brain Behav.

[13] Kułakowska A, Byfield FJ, Żendzian-Piotrowska M, Zajkowska JM, Drozdowski W (2014). Increased levels of sphingosine-1-phosphate in cerebrospinal fluid of patients diagnosed with tick-borne encephalitis. J Neuroinflamm.

[14] Du Y, Mi Z, Xie Y, Lu D, Zheng H (2011). Insights into the molecular basis of tick-borne encephalitis from multiplatform metabolomics. PLoS Negl Trop Dis.

[15] EUR-Lex-32012D0340-EN-EUR-Lex. https://eur-lex.europa.eu/eli/dec_impl/2012/340/oj. Accessed 7 Feb 2022.

[16] Lee SG, Wang T, Vance TM, Hubert P, Kim DO (2017). Validation of analytical methods for plasma total antioxidant capacity by comparing with urinary 8-isoprostane level. J Microbiol Biotechnol.

[17] Christie WW. Preparation of ester derivatives of fatty acids for chromatographic analysis. In: Christie WW, editor. Advances in lipid methodology-Two. Oily Press; 1993. p. 69–111.

[18] Luo XP, Yazdanpanah M, Bhooi N, Lehotay DC (1995). Determination of aldehydes and other lipid peroxidation products in biological samples by gas chromatographymass spectrometry. Anal Biochem.

[19] Fam SS, Murphey LJ, Terry ES, Zackert WE, Chen Y (2002). Formation of highly reactive A-ring and J-ring isoprostane-like compounds (A4/J4-neuroprostanes) in vivo from docosahexaenoic acid. J Biol Chem.

[20] Luque-Córdoba D, Calderón-Santiago M, de Castro MDL, Priego-Capote F (2018). Study of sample preparation for determination of endocannabinoids and analogous compounds in human serum by LC-MS/MS in MRM Mode. Talanta.

[21] Watkins BA, Kim J, Kenny A, Pedersen TL, Pappan KL (2016). Circulating levels of endocannabinoids and oxylipins altered by dietary lipids in older women are likely associated with previously identified gene targets. Biochim Biophys Acta.

[22] Hnasko R, Lin A, McGarvey JA, Stanker LH (2011). A rapid method to improve protein detection by indirect ELISA. Biochem Biophys Res Commun.

[23] Lee BWL, Ghode P, Ong DST (2019). Redox regulation of cell state and fate. Redox Biol.

[24] Griffiths HR, Gao D, Pararasa C (2017). Redox regulation in metabolic programming and inflammation. Redox Biol.

[25] Belikov AV, Schraven B, Simeoni L (2015). T cells and reactive oxygen species. J Biomed Sci.

[26] Pohanka M (2013). Role of oxidative stress in infectious diseases: a review. Folia Microbiol.

[27] Ingram S. Regulation of oxidoreductase enzymes during inflammation. A thesis submitted to the University of Brighton and the University of Sussex for a Programme of Study Undertaken at the Brighton and Sussex Medical School for the Degree of Doctor of Philosophy. 2018.

[28] Dobrzyńska M, Moniuszko-Malinowska A, Skrzydlewska E (2023). Metabolic response to CNS infection with flaviviruses. J Neuroinflammation.

[29] Raefsky SM, Furman R, Milne G, Pollock E, Axelsen P (2018). Deuterated polyunsaturated fatty acids reduce brain lipid peroxidation and hippocampal amyloid β-peptide levels, without discernable behavioral effects in an APP/PS1 mutant transgenic mouse model of Alzheimer’s disease. Neurobiol Aging.

[30] Palus M, Bílý T, Elsterová J, Langhansová H, Salát J (2014). Infection and injury of human astrocytes by tick-borne encephalitis virus. J Gen Virol.

[31] Lopes Pires ME, Clarke SR, Marcondes S, Gibbins JM (2017). Lipopolysaccharide potentiates platelet responses via toll-like receptor 4-stimulated Akt-Erk-PLA2 signalling. PLoS ONE.

[32] Joshi V, Venkatesha SH, Ramakrishnan C, Nanjaraj Urs AN, Hiremath V (2016). Celastrol modulates inflammation through inhibition of the catalytic activity of mediators of arachidonic acid pathway: secretory Phospholipase A 2 Group IIA, 5-Lipoxygenase and Cyclooxygenase-2. Pharmacol Res.

[33] Leu GZ, Lin TY, Hsu JTA (2004). Anti-HCV activities of selective polyunsaturated fatty acids. Biochem Biophys Res Commun.

[34] Lee SMY, Gai WW, Cheung TKW, Peiris JSM (2011). Antiviral effect of a selective COX-2 inhibitor on H5N1 infection in vitro. Antiviral Res.

[35] Karu N, Kindt A, Lamont L, van Gammeren AJ, Ermens AAM (2022). Plasma oxylipins and their precursors are strongly associated with COVID-19 Severity and with immune response markers. Metabolites.

[36] Finn DP, Haroutounian S, Hohmann AG, Krane E, Soliman N (2021). Cannabinoids, the endocannabinoid system, and pain: a review of preclinical studies. Pain.

[37] Sakin YS, Dogrul A, Ilkaya F, Seyrek M, Ulas UH (2015). The effect of FAAH, MAGL, and Dual FAAH/MAGL inhibition on inflammatory and colorectal distension-induced visceral pain models in rodents. Neurogastroenterol Motil.

[38] Krishnan G, Chatterjee N (2014). Endocannabinoids affect innate immunity of muller glia during HIV-1 tat cytotoxicity. Mol Cell Neurosci.

[39] Di Paola M, Bonechi E, Provensi G, Costa A, Clarke G (2018). Oleoylethanolamide treatment affects gut microbiota composition and the expression of intestinal cytokines in Peyer’s patches of mice. Sci Rep.

[40] Leghmar K, Cenac N, Rolland M, Martin H, Rauwel B (2015). Cytomegalovirus infection triggers the secretion of the PPARγ agonists 15-hydroxyeicosatetraenoic acid (15-HETE) and 13-hydroxyoctadecadienoic acid (13-HODE) in human cytotrophoblasts and placental cultures. PLoS ONE.

[41] Biagini D, Franzini M, Oliveri P, Lomonaco T, Ghimenti S (2022). MS-based targeted profiling of oxylipins in COVID-19: a new insight into inflammation regulation. Free Radic Biol Med.

[42] Prasad R, Giri S, Singh AK, Singh I (2008). 15-deoxy-delta12, 14-prostaglandin J2 attenuates endothelial-monocyte interaction: implication for inflammatory diseases. J Inflamm.

[43] Brandt SL, Klopfenstein N, Wang S, Winfree S, McCarthy BP (2018). Macrophage-derived LTB4 promotes abscess formation and clearance of *Staphylococcus aureus* skin infection in mice. PLoS Pathog.

[44] Widegren H, Andersson M, Borgeat P, Flamand L, Johnston S (2011). LTB4 increases nasal neutrophil activity and conditions neutrophils to exert antiviral effects. Respir Med.

[45] Li XJ, Fu HY, Yi WJ, Zhao YJ, Wang J (2015). Dual role of leukotriene B4 receptor type 1 in experimental sepsis. J Surg Res.

[46] Sorgi CA, Soares EM, Rosada RS, Bitencourt CS, Zoccal KF (2020). Eicosanoid pathway on host resistance and inflammation during Mycobacterium tuberculosis infection is comprised by LTB4 reduction but not PGE2 increment. Biochim Biophys Acta Mol Basis Dis.

[47] Apostu D, Lucaciu O, Mester A, Benea H, Oltean-Dan D (2019). Cannabinoids and bone regeneration. Drug Metab Rev.

[48] Lu HC, Mackie K (2021). Review of the endocannabinoid system. Biol Psychiatry Cogn Neurosci Neuroimaging.

[49] Han X, Wang T, Zhang J, Liu X, Li Z (2015). Apolipoprotein CIII regulates lipoprotein-associated phospholipase A2 expression via the MAPK and NFkB pathways. Biol Open.

[50] Correa F, Docagne F, Mestre L, Clemente D, Hernangómez M (2009). A Role for CB2 receptors in anandamide signalling pathways involved in the regulation of il-12 and il-23 in microglial cells. Biochem Pharmacol.

[51] Borrelli F, Romano B, Petrosino S, Pagano E, Capasso R (2015). Palmitoylethanolamide, a naturally occurring lipid, is an orally effective intestinal anti-inflammatory agent. Br J Pharmacol.

[52] Akbari N, Ostadrahimi A, Tutunchi H, Pourmoradian S, Farrin N (2022). Possible therapeutic effects of boron citrate and oleoylethanolamide supplementation in patients with COVID-19: a pilot randomized, double-blind, clinical trial. J Trace Elem Med Biol.

[53] Flannery LE, Kerr DM, Hughes EM, Kelly C, Costello J (2021). N-acylethanolamine regulation of TLR3-induced hyperthermia and neuroinflammatory gene expression: a role for PPARα. J Neuroimmunol.

[54] Lucaciu O, Aghiorghiesei O, Petrescu NB, Mirica IC, Benea HRC (2021). In quest of a new therapeutic approach in COVID-19: the endocannabinoid system. Drug Metab Rev.

